# An Assist for Cognitive Diagnostics in Soccer: Two Valid Tasks Measuring Inhibition and Cognitive Flexibility in a Soccer-Specific Setting With a Soccer-Specific Motor Response

**DOI:** 10.3389/fpsyg.2022.867849

**Published:** 2022-03-31

**Authors:** Lisa Musculus, Franziska Lautenbach, Simon Knöbel, Martin Leo Reinhard, Peter Weigel, Nils Gatzmaga, Andy Borchert, Maximilian Pelka

**Affiliations:** ^1^Department of Performance Psychology, Institute of Psychology, German Sport University Cologne, Cologne, Germany; ^2^Sport Psychology, Institute of Sport Science, Humboldt-Universität zu Berlin, Berlin, Germany; ^3^Institute for Sport Psychology and Sport Pedagogy, Leipzig, Germany; ^4^Sport Psychology and Research Methods, Institute of Sports Science, University of Tübingen, Tübingen, Germany; ^5^Umbrella Sofware GmbH, Leipzig, Germany; ^6^Institute for Applied Training Science, Leipzig, Germany; ^7^RasenBallsport Leipzig GmbH, Leipzig, Germany; ^8^FC Bayern München AG, Munich, Germany

**Keywords:** executive functions, elite youth soccer, talent identification, talent development, diagnostics

## Abstract

In professional soccer, players, coaches, and researchers alike recognize the importance of cognitive skills. Research addressing the relevance of cognitive skills has been based on the cognitive component skills approach (i.e., general cognitive processes) or the expert performance approach (i.e., sport-specific cognitive processes). Our project aimed to combine the strengths of both approaches to develop and validate cognitive tasks measuring inhibition and cognitive flexibility in a soccer-specific setting with a soccer-specific motor response. In the main study 77 elite youth soccer players completed a computerized version of the standard flanker and number–letter tasks as well as flanker and number–letter tasks requiring a soccer-specific motor response (i.e., pass) in a soccer-specific setting (i.e., the SoccerBot360). Results show good reliability for both tasks. For the SoccerBot360 number–letter task, switch effects for response times and accuracy and acceptable convergent validity were shown. A flanker effect for response time but not accuracy was apparent. Due to no acceptable convergent validity, the flanker task was revised (i.e., adaptation of stimuli) and 63 adult soccer players participated in a follow-up validation study in the SoccerBot100. The revised flanker task showed the flanker effect for response time, but not for accuracy. However, acceptable convergent validity for response time was present. Thus, the soccer-specific number–letter and to some extent the soccer-specific flanker task show potential to be used as a valid cognitive diagnostic tool by soccer clubs.

## Key points

- We developed tasks that allow to measure inhibition and cognitive flexibility in a soccer-specific setting with a soccer-specific motor response.
- We validated these cognitive tasks in two studies (*N* = 77, *N* = 63).
- We provide tasks for cognitive diagnostics in a soccer-specific setting with motor responses in the field.

## Introduction

Cognitive skills are important for soccer performance, recognized by researchers, applied sport psychologists, coaches, and players alike as a “crucial part of the modern game” (Perarnau, 2016, p. 124). That cognitive skills affect sports performance has also been supported by scientific literature (Kalén et al., in press; Voss et al., 2010; Scharfen and Memmert, 2019). However, this research is rarely used as a basis for cognitive diagnostics in the field. Most of the diagnostic instruments used to assess cognition have not been theoretically derived or empirically validated (Keegan et al., 2017; Beavan, 2019; Memmert, 2019). The aim of this project was to overcome this gap by (1) developing cognitive diagnostics in a soccer-specific setting requiring a soccer-specific motor response that are at the same time standardized and more ecologically valid and by (2) validating these tasks.

### Cognition in Sport

In sports, research on cognition has mostly been based on two approaches: The cognitive component skills approach (Nougier et al., 1991) and the expert performance approach (Ericsson, 2003). Both approaches have been used to explore the expertise of athletes. The assumption is that athletes show better *general* (cognitive component skills approach) or better *sport-specific* cognitive processes (expert performance approach) than non-athletes. We argue that it is fruitful to combine the strengths of these two approaches and develop more ecologically valid, cognitive tasks applied in a soccer-specific setting requiring a soccer-specific motor response (expert performance approach) based on and showing convergent validity with well-established standardized computer-based cognitive tasks (cognitive component skill approach).

### Cognitive Component Skills Approach

Researchers focusing on the cognitive component skills approach assume that athletes outperform non-athletes in *general* cognitive functions assessed with standardized computer-based cognitive tasks and button press responses. Such tasks have typically been developed in the field of cognitive psychology and are not sport-specific (see a detailed list in Friedman et al., 2008; Diamond, 2012). General cognitive functions include executive functions, which are top-down processes that regulate thoughts and behavior (Diamond, 2012; Miyake and Friedman, 2012). There is general agreement in the literature that inhibition, working memory, and cognitive flexibility are the core executive functions (see reviews by Diamond, 2012; Miyake and Friedman, 2012). While there is an ongoing debate about the usefulness and added value of (diagnosing and training of) executive functions to improve sports performance (see e.g., Simons et al., 2016; Beavan et al., 2020b), results of meta-analyses show that athletes outperform non-athletes in these general cognitive tasks (Voss et al., 2010; Scharfen and Memmert, 2019).

From a theoretical and an applied perspective investigating inhibition and cognitive flexibility is highly relevant. *Inhibition* is the “deliberate, controlled suppression of prepotent responses” (Miyake et al., 2000: p. 57), and *cognitive flexibility* refers to “shifting back and forth between multiple tasks, operations or mental sets” (p. 55). Theoretically, the unity-diversity framework considers inhibition to reflect the common executive function (unity) and is considered to be part of all other executive functions (Miyake and Friedman, 2012; Friedman and Miyake, 2017). In other words, inhibition is necessary for cognitive flexibility and working memory (diversity) and thus, of interest for our research. From an applied perspective, the crucial impact of inhibition and cognitive flexibility on soccer performance is evident and thus, under investigation: In a dynamic soccer situation, players frequently need to control their action alternatives and inhibit or ignore irrelevant stimuli (i.e., inhibition) and be adaptive (i.e., cognitive flexibility). In particular, players have to inhibit their previously intended action by inhibiting this prepotent action in favor of an alternative, for example when a player one was intending to pass to becomes marked by an opponent. In response to the rapidly changing situational demand, players need cognitive flexibility to quickly switch between alternative action options, for example when the coach announces a change of tactics from the side-line.

Overall, regarding the relative importance of executive function, especially inhibition and cognitive flexibility are relevant due to the increasing speed of play (Wallace and Norton, 2014) and its relation to soccer expertise and performance (Vestberg et al., 2012; Huijgen et al., 2015). In particular, most recent studies highlight this positive impact of players’ inhibitory control (Sakamoto et al., 2018; Heilmann et al., 2022a) and cognitive flexibility (Vestberg et al., 2020) on soccer performance. Nevertheless, beyond these general cognitive demands, soccer requires *specific* cognitive skills that depend on the sport context. The cognitive component skills approach has been criticized for not accounting for these specific skills (Ericsson, 2003). Overall, the theoretical value and methodologically the sport specificity of tasks is the main difference between the cognitive component skills approach and the expert performance approach.

### Expert Performance Approach

In the expert performance approach, participants, mainly athletes, are tested in sport-specific cognitive tasks that are aiming to be of high ecological validity. Sport-specificity refers to the setting (e.g., being tested in a position typical for the sport; room for movement), the stimuli presented (e.g., pictures or videos of stimuli relevant to the sport), and/or the required response (e.g., moving in a similar way to when doing the sport such as intercepting movements, passing a ball).

The main assumption is that athletes of higher expertise will outperform athletes of lower expertise in sport-specific cognitive tasks. Researchers have used this approach to study the perceptual-cognitive skills involved in a range of behaviors, from gaze behavior, attention allocation, and anticipation to decision making. Research has demonstrated that athletes also outperform non-athletes in sport-specific cognitive tasks (see meta-analyses by Kalén et al., in press; Mann et al., 2007; Scharfen and Memmert, 2019). Even though the expert performance approach aims at higher ecological validity and sport specificity is considered highly valuable in studying expertise differences, tasks used in studies conducted with this approach still often *lack* a*-sport-specific response* (e.g., show pass direction with a finger or by pressing a key or button; Voss et al., 2010). This is crucial as meta-analyses emphasize the crucial impact of sport-specific stimuli and responses (Kalén et al., in press; Mann et al., 2007).

Presenting sport-specific settings and requiring sport-specific motor responses is nowadays possible through the technological advances in the field of cognitive diagnostics and training. Tools such as Footbonaut (Saal and Fiedler, 2014), Helix (Kittelberger, 2018), or the SoccerBot (Heilmann et al., 2021), provide new possibilities to assess cognition in (more) ecologically valid settings, which is attractive to coaches and players. Following this argument, a recent study used the SoccerBot360 to administer cognitive tests requiring soccer-specific responses (i.e., passing a ball; Heilmann et al., 2021). However, the study has methodological limitations such as the low amount of trials used (e.g., 6 trials for an anticipation test). This is why the reliability and robustness of the data and the conclusions drawn remain questionable and preliminary. Thus, in the current project, we aimed to overcome limitations trough a theory-driven approach to develop cognitive tasks in a soccer-specific setting including a soccer-specific response and by systematically testing their construct validity. In particular, we combined the strengths of the two cognitive approaches: That is, we focused on the assessment of executive functions based on well-established computerized tasks (cognitive component skills approach). In addition, inspired by the strength of the expert performance approach, we aimed to develop more sport-specific, more ecologically valid inhibition, and cognitive flexibility tasks: We developed sport-specific cognitive tasks (i.e., setting and response by passing a ball) which could be used for cognitive diagnostics in soccer-specific settings in research and in the field in the future.

### The Present Project

We conducted two studies. For the first study, soccer players were recruited to perform both general and soccer-specific inhibition and cognitive flexibility tasks (flanker and number–letter tasks). Soccer-specific means that the tasks were conducted in a setting, in which participants were standing and responding to stimuli by executing soccer-specific responses, namely pass to goals. We tested adolescent soccer players from a professional youth academy because the inhibition and cognitive flexibility reach adult level during adolescence (Huizinga et al., 2006; Best and Miller, 2010).

We expected the participants to show a flanker effect and a switch effect in the soccer-specific cognitive tasks that would be comparable to those in the general versions of the tasks assessing inhibition and cognitive flexibility, respectively (Hypothesis 1). In terms of convergent validity, we expected to see a positive relationship between the general, non-sport-specific tasks and the tasks with a soccer-specific setting and a soccer-specific response modality for both inhibition and cognitive flexibility (Hypothesis 2).

Moreover, in a more exploratory fashion, we examined the relationship between inhibition and cognitive flexibility. Based on the unity/diversity framework (Miyake and Friedman, 2012; Friedman and Miyake, 2017, p. 87) that describes the relations between executive functions as separable but “not completely independent,” we expected that inhibition and cognitive flexibility are positively correlated (Hypothesis 3). In detail, we assumed that congruent trials in the flanker task would be related to no-switch trials in the number–letter task and that incongruent trials would be related to switch trials for the general tasks and the soccer-specific versions of the tasks.

## Materials and Methods: Study 1 (Validation)

### Participants: Study 1 (Validation)

*A priori* analysis in G*Power (Erdfelder et al., 2009) revealed a required sample size of a minimum of 63 participants [ρ H1 = 0.4, α = 0.05, 1−β = 0.95; the effect size estimate is based on previous work on executive functions by Huizinga et al. (2006)]. A total of 77 male soccer players from the youth academy of a German first division soccer club participated in the study. They were born between 2001 and 2005 (*M*_*age*_ = 15.7 years, *SD* = 1.3) and belonged to the U15, U16, U17, and U19 youth teams. We selected the U15 as the youngest team to participate because inhibition and cognitive flexibility reach adult-level by age 15 (Huizinga et al., 2006; Best and Miller, 2010). On average, participants had played soccer for 10.34 years (*SD* = 2.4) and practiced 10.11 h/week (*SD* = 2.69). At the time of data collection (July 2019), their teams were playing at the top level of their respective age group and the players had been part of a professional youth academy for an average of 4.5 years (*SD* = 2.5). Prior to the investigation the club obtained written informed consent from the participants and their legal guardians. The study was carried out in accordance with the Declaration of Helsinki and approved by the ethics committee of the German Sport University Cologne (Number 056/2019).

### Materials: Study 1 (Validation)

#### Instruments to Measure General and Soccer-Specific Cognition

To measure general inhibition and cognitive flexibility, we presented the computerized cognitive tasks on a 15-in. flat-screen monitor (1,280 × 960 pixels at 60 Hz) at a viewing distance of approximately 60 cm, using Inquisit 5 (2018). Participants were asked to press the correct key depending on the task. We decided to cover the relevant keys (“E” and “I”) with neutral colors (i.e., white and black stickers) to make the tasks as clear as possible.

To measure inhibition and cognitive flexibility in a soccer-specific manner, the general computerized cognitive task was transferred to the SoccerBot360 by Umbrella Software. The SoccerBot360 is a circular training device with a diameter of 10 m that provides for a 90-m^2^ field surrounded by a 32-segment wall, each segment 1-m wide and 2.5-m high, serving as a projection area for the training content and against which played balls can be kicked (see Supplementary Figure 1). Six high-definition projectors, providing a 360° experience, project the training content. An integrated high-speed camera enables the recording of parameters such as response time, processing time, and accuracy. The playing field’s ground consists of artificial grass. Thereby, the SoccerBot360 provides a soccer-specific setting by projecting footage of a 360° environment (e.g., a soccer field). Additionally, it can be programmed to provide soccer-specific stimuli (e.g., players, goals) that we used for the flanker task and it enables the measurement of a soccer-specific response (e.g., response time and accuracy when hitting a certain target; see Supplementary Material for details).

##### Inhibition

**FIGURE 1:**
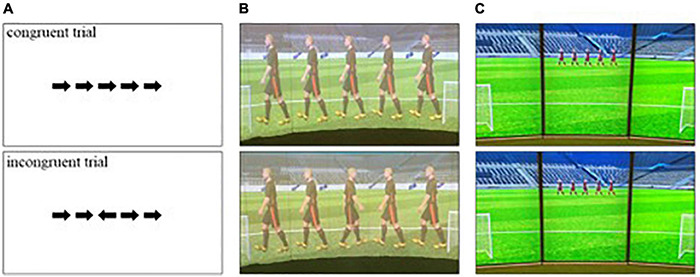
Flanker task for the computerized **(A)**, adapted for the SoccerBot360 **(B)** and after revision in SoccerBot100 **(C)**. Images of SoccerBot reproduced with permission from Umbrella Software.

An arrow version of the flanker task (Eriksen and Eriksen, 1974) was used to measure general and sport-specific inhibition. It is a commonly used and robust cognitive task (Hedge et al., 2018). For the computerized task, the stimuli were five black arrows on a white background presented on a computer screen. The middle arrow was identified as the target arrow. The arrows either pointed all in the same direction (congruent trials) or the middle arrow only pointed in the opposite direction (incongruent trials), as illustrated in Figure 1A. Participants were asked to respond only to the target arrows by pressing either the white key (the covered “E” key) when the target arrow pointed to the left or the black key (the covered “I” key) when the target arrow pointed to the right as quickly and accurately as possible, thereby inhibiting the flanking stimuli.

For the flanker task developed and used in the SoccerBot, setting, response, and stimuli were soccer-specific: Players were standing in a soccer field; They had to respond by passing a ball; And as stimuli, five soccer players were presented from the side (Figure 1B). In accordance with the general flanker task, the target player was in the middle with two players on each side. Depending on the direction in which the target player faced, participants were asked to kick the ball into the left or right goal presented on either side of the players. From here on, we refer to this task as soccer-specific flanker task.

Requiring participants to process both flankers and targets at the same time is the main idea of the flanker task (Verbruggen et al., 2004). Because congruent flankers activate the correct response, but incongruent flankers prime an incorrect response (Ridderinkhof and van der Molen, 1995), response times are expected to be prolonged in the incongruent trials, where participants must inhibit the incorrect response (Coles et al., 1985). The so-called flanker effect is determined by the participants’ individual difference between incongruent and congruent mean response time for correct trials. The lower the flanker effect, the better the inhibitory control.

Further details of the general and soccer-specific flanker task are presented in Supplementary Table 1 (see Supplementary Table 2 for justification based on previous studies). It should be noted that we used twice as many congruent trials as incongruent trials because using fewer incongruent trials has been shown to ensure higher demand on inhibitory control (for flanker task, see Krenn et al., 2018).

##### Cognitive Flexibility

**FIGURE 2:**
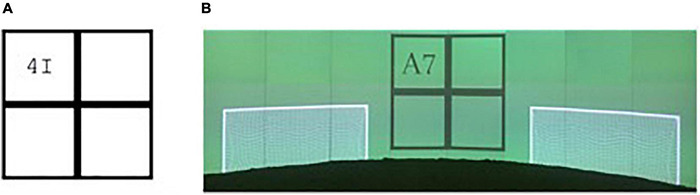
Number-letter task in the computerized version **(A)** and adapted for the SoccerBot360 **(B)**. Images of SoccerBot reproduced with permission from Umbrella Software.

Cognitive flexibility was assessed using a version of the number–letter task as adapted from Rogers and Monsell (1995) by Miyake et al. (2000). For the computerized as well as the SoccerBot360 task, participants were presented with a 2 × 2 matrix (see Figure 2). In each trial, a number–letter pair (e.g., 4I or A7) appeared in one of four quadrants. The numbers were even (2, 4, 6, 8) or odd (3, 5, 7, 9). The letters were vowels (A, E, I, O) or consonants (G, K, M, R). When the number–letter pair appeared in one of the top two quadrants, participants were asked to focus on the letter, pressing the white “E” key when the letter was a consonant and the black “I” key when it was a vowel. When the number–letter pair appeared in one of the bottom two quadrants, participants were asked to focus on the number, pressing “E” when the number was even and “I” when the number was odd.

For the number-letter task developed and used in the SoccerBot, setting, and response were soccer-specific: Players were standing in a soccer field and had to respond by passing a ball to one of the goals depending on the presented stimulus. From here on, we refer to this task as soccer-specific number-letter task.

The number–letter task is designed to assess how people switch between two different sets of rules depending on the context (i.e., presented stimuli and stimuli location). Whereas response time is typically slower for “switch trials” (i.e., change in rule: switch from number to letter or vice versa) compared to “no-switch trials” (i.e., no change in rule: continuing number or letter task), the reverse is true for accuracy, that is, fewer correct switch trials compared to no-switch trials (Monsell, 2003). This so-called switch cost or switch effect is therefore calculated as the difference in response times and accuracy between the switch trials and the no-switch trials. Participants with lower shift costs are thought to have higher cognitive flexibility.

Further details of the general and soccer-specific number–letter task are presented in Supplementary Table 1 (see Supplementary Table 2 for justification based on previous studies).

#### Control Variables: Motivation, Physical Exertion, and Fun

Motivation of the participants was assessed as a control variable. For this purpose, they answered the question “How motivated are you at this moment?” on a visual analog scale (VAS; Crichton, 2001) of 0 (*not at all*) to 100 (*highly*) before performing the computerized and SoccerBot360 tasks. The scale was presented in digital form on a tablet.

In addition, perceived physical exertion of the participants was assessed using the 15-point Borg scale for ratings of perceived exertion (Borg, 1970). This assessment was also made before participants performed the tasks to control for potential increased physical load during the experiment.

Finally, we assessed perceived fun regarding the tasks. Players were asked to answer the question “How much fun did you have doing the current task?” on a VAS scale of 0 (*none*) to 100 (*a lot*).

### Procedure: Study 1 (Validation)

Our first step was to develop the tasks to measure soccer-specific inhibition and cognitive flexibility. This was accomplished in collaboration with the soccer club academy’s sport psychologists and sports scientists. Programmers of the Umbrella Software Company then implemented this task for the SoccerBot360.

Next, we ran a pilot study with three players and two coaches at the participating academy. They performed both tasks and gave critical feedback especially for the soccer-specific tasks. Considering their experience with the SoccerBot360 and their knowledge and competencies in soccer, we reduced the number of trials (flanker task: 144–108 test trials; number–letter task: 128–112 test trials) and the response stimulus interval was reduced from 2,000 to 1,000 ms.

For the final experiment, players were informed about the study by their coaches and sports psychologists prior to data collection during the teams’ preseason preparation in 2019 and written informed consent was obtained before testing from the participants or their legal guardians by the club. The experiment was conducted in the youth soccer academy of the participating club and lasted approximately 75 min for each player. The general computerized tasks were executed in a dressing room for referees and the SoccerBot360 is in the academy’s sports hall, an approximately 2-min walk away. Two experimenters supervised the study, one overseeing the computer room and one the SoccerBot360, alternating daily.

The study followed a cross-sectional approach. First, participants were asked to fill out demographic and soccer-specific questionnaires. This was followed by the general cognitive tasks on the computer. After that, participants were asked to walk to the SoccerBot360 where they were then asked to warm up individually for 5–10 min to reduce the risk of injuries, before starting the tasks in the SoccerBot360. All tasks on the computer and the SoccerBot360 were presented in a counterbalanced order. The order of the versions (i.e., first computer, second SoccerBot360) was set for logistical reasons but more importantly, in order to familiarize the players with each task and reduce the number of practice trials, and therefore the physical load, in the SoccerBot360, especially for the number–letter task. Before both the computerized cognitive task and the soccer-specific cognitive tasks participants were asked to fill out the Borg scale to assess perceived physical exertion and the VAS assessing motivation. In the end, players were asked to state how much fun they had had and were thanked for their participation.

### Data Preparation: Study 1 (Validation)

For both the flanker and the number–letter task, all trials with incorrect responses were excluded from the analysis (2.38% computerized flanker task; 0.10% soccer-specific flanker task; 9.11% computerized number–letter task; 5.34% soccer-specific number–letter task). In a second filter for the computerized task, all trials with response times lower than 200 ms or higher than 1,750 ms in the flanker tasks (0.009%) and lower than 200 ms or higher than 3,000 ms in the number–letter tasks (2.55%) were excluded to account for extreme results (e.g., Lautenbach et al., 2016). For the soccer-specific tasks, the same filter (flanker task: 0.39%; number–letter task: 1.28%) was used but with 400 ms as the lower bound, assuming longer response times for whole-body actions that take into account the visuomotor interval and the time interval between take-off of the kicking foot and ball contact (as also described by Morya et al., 2003). A third filter excluded response times ± 3 *SD* from the individual mean (1.22% computerized flanker task; 0.95% soccer-specific flanker task; 0.87% computerized number–letter task; 1.22% soccer-specific number–letter task).

Overall, three players had to be excluded because of incomplete data sets (one in the computerized tasks, one in the soccer-specific tasks, one for both the computerized and the soccer-specific tasks). For the flanker task, an additional three participants were excluded because of their overall error rates in the first filter, either on the computerized task (> 15%) or on the soccer-specific task (> 10%; see Verbruggen et al., 2004). For the number–letter task, nine participants were excluded because they had an average accuracy (i.e., percentage of correct trials) of 70% or less in either the computerized (*n* = 4) or the soccer-specific (*n* = 5) task (in accordance with Adrover-Roig et al., 2012). In total, analyses of the flanker task include data of 72 participants and analyses of the number–letter task include data of 66 participants. Complete data sets were available for 63 participants.

### Data Analyses: Study 1 (Validation)

The dependent variables were first checked for normality and outliers. For the soccer-specific flanker task in the SoccerBot360 accuracy and response time parameters in the incongruent and congruent conditions were not normally distributed. Also, for the number-letter task, no-switch trials in the computerized version and switch trials, no-switch trials and switch effect were not normally distributed in the soccer-specific number–letter task. However, recent literature shows the relative robustness of analysis of variance (ANOVA) against violations of the normal distribution, so we applied parametric tests (see Wilcox, 2011; Blanca et al., 2017).

One outlier (*M* ± 3 × *SD*) was detected for the number–letter task in the no-switch trials of both the computerized general and the soccer-specific task. Additionally, one outlier was detected only for the no-switch trials in the soccer-specific task. All data were analyzed using SPSS Statistics, version 25. Initially, the level of significance was set at *p* < 0.05 for all analyses. First, to control for potential influences of motivation and perceived exhaustion, we checked whether there was a difference in motivation or perceived exhaustion prior to the computerized and soccer-specific tasks by running two paired *t*-tests. If differences were found, we followed up by calculating Pearson correlations with the relevant inhibitory (congruent, incongruent) and cognitive flexibility (switch, no-switch) variables.

To test Hypothesis 1 for inhibition as well as cognitive flexibility, we ran two one-factorial multivariate analyses of variance (MANOVAs), in which we tested the main effect of condition (congruent vs. incongruent and switch vs. no-switch) on accuracy and response time in both the general, computerized task and the soccer-specific tasks. To test Hypothesis 2, we calculated Pearson correlations between inhibition (congruent, incongruent trials) and cognitive flexibility (switch, no-switch) for the general, computerized tasks and the soccer-specific tasks, respectively. Finally, we ran a dependent *t*-test to assess the fun participants perceived during the two versions of the tasks.

## Results: Study 1 (Validation)

Statistical analyses indicated the same pattern of results when outliers were included and thus all analyses are reported including the outliers. The descriptive statistics for response times and accuracy for the computerized general and soccer-specific versions of the flanker task and number–letter task are shown in Table 1. Reliability, assessed via split-half reliability (coefficient *r*) and Cronbach’s Alpha for response time parameters, show high values for both computerized general (flanker, congruent: *r* = 0.89; α = 0.93, incongruent: *r* = 0.86; α = 0.89, number-letter, switch: *r* = 0.82; α = 0.95, no-switch: *r* = 0.82; α = 0.93) and soccer-specific tasks (flanker, congruent: *r* = 0.98; α = 0.96, incongruent: *r* = 0.96; α = 0.95, number-letter, switch: *r* = 0.92; α = 0.94, no-switch: *r* = 0.90; α = 0.95).

**TABLE 1:** Descriptive statistics for the general, computerized and the soccer-specific versions of the tasks measuring inhibition (flanker; *n* = 72) and cognitive flexibility (number–letter; *n* = 66).

Task	Descriptive statistics			
	*M*	*SD*	Min	Max
**Study 1: Number–letter task**				
RT, computerized, no-switch trials (ms)	897.56	202.77	653.56	2.046.09
RT, computerized, switch trials (ms)	1.411.69	262.06	806.21	2,061.25
Accuracy, computerized (%)	90.11	6.67	72.66	100.00
RT, soccer-specific, no-switch trials (ms)	1.229.13	232.16	871.76	2.116.49
RT, soccer-specific, switch trials (ms)	1.392.21	269.03	939.89	2.086.85
Accuracy, soccer-specific (%)	94.30	5.39	75.89	100.00
**Study 1: Flanker task**				
RT, computerized, congruent trials (ms)	403.46	34.65	328.87	505.41
RT, computerized, incongruent trials (ms)	425.53	35.36	342.90	515.77
Accuracy, computerized (%)	94.97	2.48	91.15	100.00
RT, soccer-specific, congruent trials (ms)	990.10	153.59	800.82	1.595.18
RT, soccer-specific, incongruent trials (ms)	1.001.29	150.73	807.39	1.631.77
Accuracy, soccer-specific (%)	99.88	0.005	98.61	100.00
**Study 2: Revised flanker task**				
RT, computerized, congruent trials (ms)	393.42	53.59	603.80	308.60
RT, computerized, incongruent trials (ms)	421.49	49.33	330.28	621.17
Accuracy, computerized (%)	98.52	1.83	93.06	100.00
RT, soccer-specific, congruent trials (ms)	997.93	154.09	729.55	1402.72
RT, soccer-specific, incongruent trials (ms)	1027.66	161.24	717.72	1517.44
Accuracy, soccer-specific (%)	99.94	0.32	98.15	100.00

*RT, response time.*

### Control Variables (Motivation, Perceived Exhaustion, and Perceived Fun)

There was no significant difference in motivation prior to the computerized general (*M* = 77.51, *SD* = 21.44) and soccer-specific (*M* = 73.73, *SD* = 21.96) tasks, *t*(62) = 1.7, *p* = 0.094, *d* = 0.166. However, the perceived exhaustion was significantly higher prior to the soccer-specific task in the SoccerBot360 (*M* = 13.32, *SD* = 2.07) in comparison to the general task on the computer (*M* = 11.17, *SD* = 3.05), *t*(62) = 4.99, *p* < 0.001, *d* = 0.825. A Pearson correlation, however, did not show any significant correlations between perceived exhaustion and performance in the computerized (*r* < 0.145, *p* > 0.257) or soccer-specific (*r* < 0.200, *p* > 0.116) tasks. Finally, elite youth soccer players found the soccer-specific tasks in the SoccerBot360 to be significantly more fun (*M* = 75.86, *SD* = 21.6) than the computerized tasks (*M* = 66.16, *SD* = 21.13), *t*(62) = 3.457, *p* = 0.001, *d* = 0.454.

### Inhibition

#### Flanker Effect (Hypothesis 1)

For the flanker task, the 2 (Task Version: computerized vs. soccer-specific) × 2 (Trial: congruent vs. incongruent) repeated-measures MANOVA showed a significant multivariate main effect of task version, Wilks’s Λ = 0.05, *F*(2, 70) = 610.20, *p* < 0.001, η*_*p*_*^2^ = 0.95, and trial, Wilks’s Λ = 0.38, *F*(2, 70) = 58.02, *p* < 0.001, η*_*p*_*^2^ = 0.62, as well as a significant interaction of task version and trial, Wilks’s Λ = 0.68, *F*(2, 70) = 16.30, *p* < 0.001, η*_*p*_*^2^ = 0.32, on both dependent variables. Following up the significant multivariate main effect with univariate ANOVAs showed that the main effects of task version and trial were significant for both response time, task version: *F*(1, 71) = 1082.50, *p* < 0.001, η*_*p*_*^2^ = 0.94; trial: *F*(1, 71) = 92.95, *p* < 0.001, η*_*p*_*^2^ = 0.57, and accuracy, task version: *F*(1, 71) = 116.91, *p* < 0.001, η*_*p*_*^2^ = 0.62; trial: *F*(1, 71) = 30.03, *p* < 0.001, η*_*p*_*^2^ = 0.30. Further, the Task Version × Trial interaction was also significant for response time, *F*(1, 71) = 14.43, *p* < 0.001, η*_*p*_*^2^ = 0.17, and accuracy, *F*(1, 71) = 21.73, *p* < 0.001, η*_*p*_*^2^ = 0.23.

To scrutinize the significant interaction in the flanker task, we conducted further repeated-measures ANOVAs for each task version separately. For the computerized general flanker task, the repeated-measures ANOVA showed a significant main effect of trial for response time, *F*(1, 71) = 122.797, *p* > 0.001, η*_*p*_*^2^ = 0.634, and accuracy, *F*(1, 71) = 27.013, *p* > 0.001, η*_*p*_*^2^ = 0.276. Also for the soccer-specific flanker task in the SoccerBot360, a main effect of trial for response time was detected, *F*(1, 71) = 20.602, *p* < 0.001, η*_*p*_*^2^ = 0.225, indicating that participants needed significantly longer to respond to incongruent trials in comparison to congruent trials (i.e., flanker effect). However, no significant main effect was found for accuracy, *F*(1, 71) = 0.724, *p* = 0.389, η*_*p*_*^2^ = 0.010, indicating that participants responded correctly most of the time (congruent: 99.92%; incongruent: 99.85%) independent of the type of trial.

#### Convergent Validity (Hypothesis 2)

For the computerized and the soccer-specific flanker tasks, correlational analyses revealed no significant correlations for response time (congruent trials: *r* = 0.142, *p* = 0.234; incongruent trials: *r* = 0.190, *p* = 0.110) or accuracy (congruent trials: *r* = −0.022, *p* = 0.855; incongruent trials: *r* = −0.118, *p* = 0.322). This indicates that the newly developed soccer-specific flanker task is not related to the general, computerized flanker task.

### Cognitive Flexibility

#### Switch Effect (Hypothesis 1)

For the number–letter task, the 2 (Task Version: computerized vs. soccer-specific) × 2 (Trial: congruent vs. incongruent) repeated-measures MANOVA showed a significant multivariate main effect of task version, *F*(1, 64) = 23.56, *p* < 0.001, η*_*p*_*^2^ = 0.42, and trial, *F*(1, 64) = 212.99, *p* < 0.001, η*_*p*_*^2^ = 0.87, as well as a significant interaction of task version and trial, *F*(1, 64) = 143.09, *p* = 0.005, η*_*p*_*^2^ = 0.82, on both dependent variables. Following up the significant multivariate main effect with univariate ANOVAs, main effects of task version and trial were significant for response time, task version: *F*(1, 65) = 33.49, *p* <0.001, η*_*p*_*^2^ = 0.34; trial: *F*(1, 65) = 376.51, *p* < 0.001, η*_*p*_*^2^ = 0.85, and accuracy, task version: *F*(1, 65) = 30.30, *p* < 0.001, η*_*p*_*^2^ = 0.32; trial: *F*(1, 65) = 137.06, *p* < 0.001, η*_*p*_*^2^ = 0.68. Further, the Task × Trial interaction was also significant for response time, *F*(1, 65) = 239.10, *p* < 0.001, η*_*p*_*^2^ = 0.85, and accuracy, *F*(1, 65) = 33.56, *p* < 0.001, η*_*p*_*^2^ = 0.68.

For the soccer-specific number–letter task in the SoccerBot360, the ANOVAs revealed a significant main effect of trial for response time, *F*(1, 65) = 128.28, *p* < 0.001, η*_*p*_*^2^ = 0.66, and accuracy, *F*(1, 65) = 23.80, *p* < 0.001, η*_*p*_*^2^ = 0.27. This indicates that participants were faster and more accurate in the no-switch trials (response time: *M* = 1.229.12, *SD* 232.16; accuracy: *M* = 95.56, *SD* = 5.35) compared to the switch trials (response time: *M* = 1.392.21, *SD* = 269.03; accuracy: *M* = 93.05, *SD* = 6.18) in the soccer-specific cognitive flexibility task.

#### Convergent Validity (Hypothesis 2)

For the computerized and soccer-specific number–letter tasks, correlational analyses revealed positive medium correlations: For response time and accuracy, the tasks were positively correlated for switch (response time: *r* = 0.534, *p* < 0.001; accuracy: *r* = 0.436, *p* < 0.001) and no-switch (response time: *r* = 0.530, *p* < 0.001; accuracy: *r* = 0.367, *p* = 0.002) trials. In sum, these correlational patterns indicate that the soccer-specific number–letter task has convergent validity with the general, computerized number–letter task.

### Relation Between General and Sport-Specific Inhibition and Cognitive Flexibility (Hypothesis 3)

The exploratory correlational analyses to investigate if the relation between inhibition and cognitive flexibility was comparable for the general, computerized task and the newly developed soccer-specific task revealed comparable correlational patterns for response time in the congruent inhibition and no-switch trials (see Table 2).

**TABLE 2:** Correlations between inhibition (measured with congruent and incongruent trials in the flanker task) and cognitive flexibility (measured with switch and no-switch trials in the number–letter task) in the general, computerized and the soccer-specific tasks.

Variable	Number–letter task	Computerized task		Soccer-specific task	
		
		Flanker Task			
		Congruent	Incongruent	Congruent	Incongruent
Response time	No-switch	0.148	0.061	0.324**	0.330**
	Switch	0.270*	0.194	0.265*	0.259*
Accuracy	No-switch	0.174	0.172	0.085	0.105
	Switch	0.205	0.205	0.036	0.251*

*p < 0.05; **p < 0.001.

## Intermediate Discussion Study 1 (Validation)

In Study 1, we tested elite youth soccer players who performed general, computerized cognitive tasks and the adapted soccer-specific tasks to assess inhibition and cognitive flexibility. For the soccer-specific cognitive tasks in the SoccerBot360, players were standing and moving with the ball (soccer-specific setting) and had to respond with a sport-specific response, namely, by passing a ball. In short, the results revealed that the soccer-specific cognitive flexibility task had acceptable convergent validity, while the soccer-specific inhibition task was not. The results will be discussed in more detail in the general discussion below. However, two potential limitations of Study 1 led to the follow-up study: First, Study 1 was conducted with elite youth soccer players, which could limit the generalizability of the results. Second, the soccer-specific inhibition task showed lacking convergent validity. This is why we revised the soccer-specific inhibition task and enrolled adult soccer players in the follow-up Study 2.

## Study 2: Follow-Up

The follow-up Study 2 aimed at modifying and further testing the convergent validity of a revised flanker task for the soccer-specific setting in a sample of adult soccer players. The soccer-specific flanker task developed and tested in our main study did not show satisfactory convergent validity. As a potential reason for these results, we identified the size of the player stimuli and the size-relation of presented stimuli and the distance of the participants to the screen. In detail, the players presented on the screen were simply too tall for the viewing distance of 5 m. Consequently, the size of the players was distinctly reduced for the soccer-specific flanker task. For this size adjustment, we calculated the optimal presentation size of the stimuli based on a realistic virtual angle of 2.5–3.7° in relation to a 5 m distance to the screen proposed by Murphy et al. (2016) see also: Loffing et al. (2011). This led to a presentation size of 38 cm height and 20 cm width for each player stimulus (see Figure 1C for the new size of player stimuli presented). Further, the number of trials was reduced by half for both tasks, especially to reduce physical load and prevent injuries as well as to avoid a decrease in motivation. Based on correlational analyses regarding the results of split-half reliability in Study 1 the reduction of trials did not affect the reliability of the tasks (computerized task congruent: *r* = 0.89, incongruent: *r* = 0.86 and soccer-specific task congruent: *r* = 0.98, incongruent: *r* = 0.96). We hypothesized, as in the main Study 1, that participants would show a flanker effect in the soccer-specific flanker task in the SoccerBot that would be comparable to the effects found in the computer-based versions of the task assessing inhibition (Hypothesis 2.1) and that the revised soccer-specific flanker task would show a positive correlational relationship to the general flanker task regarding convergent validity (Hypothesis 2.2).

### Participants: Study 2 (Follow-Up)

Based on the *a priori* G*power analysis (for exact settings see Study 1), a total of 63 male soccer players (*M*_*age*_ = 25.17, *SD*_*age*_ = 4.81) participated in the study. Only adult players with soccer experience of at least one competitive season in club football were included. Twenty-one participants used to play in a youth academy when they were younger. On average, participants had played soccer for 16.17 years (*SD* = 5.61) and practiced 4.72 h/week (*SD* = 2.69). Most players played in the 6th (*n* = 27) and 7th highest league in Germany (*n* = 13), whereas only two players played in the 9th and 10th league. Four played in the 4th highest, nine in the 5th highest league, and finally six played in the 8th highest league. Prior to the investigation, informed consent and assent for all players was received. The study was carried out in accordance with the Declaration of Helsinki.

### Material: Study 2 (Follow-Up)

To measure general inhibition, the computerized flanker task was presented in the same way as in the main Study 1. To measure inhibition soccer-specifically, the SoccerBot360 inhibition task was modified and transferred to the SoccerBot100. The modified task presented five players with a height of 38 cm on a 1-m wide segment (see Figure 1C). The SoccerBot100 is a smaller version of the SoccerBot360 with a smaller field but with walls for projections. The training content is shown on 7 full HD screens with a viewing angle of 100°. The playing ground is artificial grass. The starting point where the participants pass and control the ball is 5 m away from the screen which is identical to the Soccerbot360.

### Data Preparation and Analyses: Study 2 (Follow-Up)

Data preparation and analyses followed the exact protocol of Study 1. Accordingly, the analyses include data of 63 participants. Two outliers (*M* ± 3 × *SD*) were detected in the congruent trials of the computerized task. Additionally, two outliers were detected in the incongruent trials of the computerized task as well as one outlier in the incongruent soccer-specific trials in the SoccerBot100.

## Results: Study 2 (Follow-Up)

### Control Variables (Motivation, Perceived Exhaustion, and Perceived Fun)

No significant difference in motivation was detected prior to the computerized and soccer-specific tasks, *t*(62) = 0.861, *p* = 3.93, *d* = 0.101 as well as for perceived exhaustion, *t*(62) = 1.332, *p* = 0.188, *d* = 0.176. For the fun perceived, results were similar to the results of Study 1: The participating players found the SoccerBot000 soccer-specific flanker task to be significantly more fun (*M* = 85.17, *SD* = 16.609) than the computerized task (*M* = 64.32, *SD* = 25.49), *t*(62) = −6.510, *p* < 0.01, *d* = 0.969.

### Flanker Effect (Hypothesis 2.1)

Statistical analyses indicated the same pattern of results when outliers were included and thus, all analyses are reported including the outliers. The descriptive statistics for response times and accuracy for the computerized and revised soccer-specific versions of the flanker task are shown in Table 1. Reliability, assessed via split-half reliability and Cronbach’s alpha for response time parameters, show high values for both computerized (congruent: *r* = 0.91; α = 0.96, incongruent: *r* = 0.90; α = 0.91) and soccer-specific task (congruent: *r* = 0.94; α = 0.97, incongruent: *r* = 0.92; α = 0.97).

For the revised flanker task, the 2 (Task Version: computerized vs. soccer-specific) × 2 (Trial: congruent vs. incongruent) repeated-measures MANOVA showed a significant multivariate main effect of task version, Wilks’s Λ = 0.042, *F*(2, 61) = 679.89, *p* < 0.001, η*_*p*_*^2^ = 0.958, and trial, Wilks’s Λ = 0.231, *F*(2, 61) = 101.31, *p* < 0.001, η*_*p*_*^2^ = 0.769, as well as a significant interaction of task version and trial, Wilks’s Λ = 0.713, *F*(2, 61) = 12.28, *p* < 0.001, η*_*p*_*^2^ = 0.29, on both dependent variables. Following up the significant main effect with univariate ANOVAs showed that the main effect of task version was significant for response time: *F*(1, 62) = 1323.34, *p* < 0.001, η*_*p*_*^2^ = 0.96; and accuracy: *F*(1, 62) = 37.54, *p* < 0.001, η*_*p*_*^2^ = 0.38. However, the main effect of trial was significant for response time *F*(1, 62) = 186.84, *p* < 0.001, η*_*p*_*^2^ = 0.75 and for accuracy *F*(1, 62) = 21.129, *p* < 0.001, η*_*p*_*^2^ = 0.75. Further, the Task Version × Trial interaction was not significant for response time, *F*(1, 62) = 0.161, *p* = 689, η*_*p*_*^2^ = 0.01, but for accuracy, *F*(1, 62) = 24.14, *p* < 0.001, η*_*p*_*^2^ = 0.28.

To scrutinize the significant interaction in the flanker task, we conducted further repeated-measures ANOVAs for each task version separately. For the computerized flanker task, the repeated-measures ANOVA showed a significant main effect of trial for response time, *F*(1, 62) = 121.837, *p* > 0.001, η*_*p*_*^2^ = 0.663, and accuracy, *F*(1, 62) = 23.627, *p* > 0.001, η*_*p*_*^2^ = 0.276. Also for the revised soccer-specific flanker task in the SoccerBot100, a main effect of trial for response time was detected, *F*(1, 62) = 80.545, *p* < 0.001, η*_*p*_*^2^ = 0.565, indicating that participants needed significantly longer to respond to incongruent trials in comparison to congruent trials, presenting the typical flanker effect. However, no significant main effect was found for accuracy, *F*(1, 62) = 0.197, *p* = 0.658, η*_*p*_*^2^ = 0.658, indicating that participants responded correctly most of the time (congruent: 99.95%; incongruent: 99.91%) independent of the type of trial.

### Convergent Validity (Hypothesis 2.2)

For the computerized and the adjusted soccer-specific flanker task, correlational analyses revealed significant correlations for response time (congruent trials: *r* = 0.611, *p* < 0.01; incongruent trials: *r* = 0.620, *p* < 0.01) but not for accuracy (congruent trials: *r* = −0.149, *p* = 0.243; incongruent trials: *r* = 0.041, *p* = 0.749).

## Intermediate Discussion Study 2 (Follow-Up)

The follow-up Study 2 aimed to test a revised version of the soccer-specific inhibition task with adult soccer players. In Study 2, the size of players presented in the SoccerBot100 for the adjusted soccer-specific flanker task was distinctly reduced in comparison to the presentation in the main Study 1. The results showed a flanker effect for response time, with participants reacting more slowly in response to incongruent compared to congruent trials. However, no flanker effect was found for accuracy in the adjusted soccer-specific flanker task. Participants were similarly correct when responding to congruent and incongruent trials, which is contrary to general findings on the flanker task (Miyake et al., 2000) and to the results found in the general, computerized task. Thus, it seems that it was easier for participants to respond correctly in the adjusted soccer-specific flanker task, which will be discussed in detail below.

For convergent validity of the general and adjusted soccer-specific flanker inhibition task, we found strong positive correlations for response time parameters in both task conditions. Results regarding the reliability and validity of the reaction-time measure indicate that the revised version of the flanker task for the SoccerBot100 can be used to assess inhibition in adult soccer players. However, the results have to be interpreted carefully, as validity could only be detected for response time parameters so far.

For accuracy, very low variance in this revised version of the task was apparent, so that despite the reduced size of the projected stimuli (i.e., players), this might be a reason why we did not detect the expected positive correlations. In this vein, the longer times needed for motor responses could be an important aspect to consider regarding the relationship between the accuracy values of the computerized and the SoccerBot100 flanker task. Based on the memory-drum theory by Henry and Rogers (1960), stating that response times are directly related to the complexity of the response that has to be initiated, the soccer-specific response requiring players to execute a precise pass with the foot can be considered more complex and in need of more comprehensive motor preparation than a button press with the index finger, resulting in longer times until execution. Thus, in the soccer-specific setting, the players might have had more time to potentially suppress their first response tendency and to inhibit their response because the activation and movement of the corresponding muscles take longer. This might be an explanation for the low errors in the adjusted soccer-specific flanker task.

## General Discussion

In the present project, we aimed at developing and validating inhibition and cognitive flexibility tasks in a soccer-specific setting including soccer-specific responses. Thereby, it was our goal to combine the strengths of the cognitive component skills approach and the expert performance approach by assessing general executive functions in a more ecologically valid setting requiring a soccer-specific response. To evaluate the newly developed tasks, we conducted a first validation study with 72 elite youth soccer players and a follow-up study with 63 soccer players, in which a revised version of the soccer-specific flanker task was tested further.

### Soccer-Specific Inhibition Task Shows Flanker Effect but no Convergent Validity

In the present project, our soccer-specific flanker task (i.e., soccer-specific setting, stimuli, and response) presented images of players facing left or right. As expected, the results showed a flanker effect for response time, with participants reacting more slowly in response to incongruent compared to congruent trials. This seems to show that the participants needed to cognitively engage more to inhibit a response in incongruent trials (cf. Krenn et al., 2018) and is in line with the mechanism proposed for the flanker task (Eriksen and Eriksen, 1974). However, in contrast to expectations, no flanker effect was shown for accuracy. Participants were similarly correct when responding to congruent and incongruent trials, which is contrary to general findings on the flanker task (Miyake et al., 2000) and to the results found in the general, computerized task performed by the same participants in our main study. It seems that it was easier for participants to respond correctly in the soccer-specific setting task. This might be explained by the relatively large size of the players presented as stimuli, which might have made detection of direction easier. After refinements of the task regarding the size of the players in the follow-up study still no flanker effect was shown for accuracy. Here, it must be mentioned that, the motor response in the soccer-specific flanker task in the SoccerBot360 is, on the one hand, a more complex movement in comparison to pressing a button, but on the other hand, it allows participants to suppress a started action that might have been a wrong response, thereby increasing their accuracy.

For convergent validity of the general and soccer-specific flanker task, we found no relationship in the scope of the first study. A possible explanation for not finding positive correlations for response times might be that there was fairly low variance in the computerized task, as can be seen in the descriptive statistics (see Table 1) and depicted in a scatter plot (see Supplementary Figure 2). Regarding accuracy, a very low variance in the soccer-specific flanker task, potentially due to the size of the projected player stimuli, and the possibility of adapting the motor response in the SoccerBot360 more effectively might also be why we did not detect the expected positive correlations.

Given the results of the soccer-specific flanker task in the first validation study, it seemed difficult to state confidently that the newly developed task can be considered a valid measurement and used for diagnostics in the applied field. Although the typical response latency for incongruent trials in comparison to congruent trials was evident for the soccer-specific flanker task, which indicates the assumed mechanism of the flanker effect, the results did not show convergent validity with respect to the computerized task. Consequently, further refinements (e.g., decreasing the size of presented stimuli) were implemented to increase the convergent validity when comparing a general, computerized inhibition task with an adapted version in the SoccerBot100.

Following the refinements of the task, the second validation study was conducted to re-investigate convergent validity. After reducing the stimuli size, convergent validity could be established for response time parameters. This indicates that the revised soccer-specific flanker task is related to the general, non-sport-specific flanker task. Thus, the revised task can be applied to assess response time parameters reflecting inhibition. Regarding the accuracy, it seems difficult to reach values similar to the general, computerized task, which might be due to the aforementioned reasons. Along these lines, the accuracy of the SoccerBot flanker task could be assessed beyond the correct direction (i.e., pass to the left or right) decision. The accuracy of the motor response (i.e., pass) could be better captured by assessing the precision of the pass. In detail, the precision could be captured by determining whether or not the pass hit the goal (i.e., target area). Further, the goal could even be scaled in size and different target sizes could be presented across trials to obtain a scaled accuracy and precision value for each player. This would be a high added value for diagnostics of individual players and for designing individually tailored cognitive training interventions.

### Soccer-Specific Cognitive Flexibility Task Shows Convergent Validity

The soccer-specific cognitive flexibility task required participants to switch between rules and to pass a ball accordingly, eliciting a switch effect. The results demonstrate that switching between rules negatively affected both response time and accuracy, as expected. Importantly, the costs of switching between rules also affected the soccer-specific response, that is, passing the ball in the right direction. This is important because it shows that the entire motor-cognitive control system of the participants seemed to be affected.

In addition, we looked at the convergent validity of the general and the soccer-specific cognitive flexibility tasks. The results show medium correlations for both response time and accuracy for the number–letter tasks. Thus, the soccer-specific cognitive flexibility task can be considered a valid measurement that can be applied to assess whether players can cognitively switch between rules. The task can be implemented as a cognitive diagnostic instrument with soccer-specific motor response and might be used by researchers as well as sport psychologists working in soccer.

### Soccer-Specific Inhibition and Cognitive Flexibility Tasks Are Related

The relation between the two cognitive functions, that is, inhibition and cognitive flexibility, in the general computerized tasks and the soccer-specific tasks were also explored in the present project. Interestingly, while we detected only one significant correlation (for response time) within the computerized tasks, most correlations in the soccer-specific tasks in the SoccerBot360 were significant: The response times and accuracy for incongruent trials in the flanker task and switch trials in the number–letter task were significantly positively related, and the response times for congruent trials in the flanker task and no-switch trials in the number–letter task were significantly positively related. Thus, the harder it was and the longer it took for participants to inhibit a wrong response in the flanker task, the harder it was and the longer it took for them to switch between rules in the number–letter task. Even though the correlations between inhibition and cognitive flexibility are not as strong as presented in previous research (e.g., *r* = 0.77 in Friedman et al., 2011), the pattern of results is in line with the theoretical predictions of the unity–diversity framework (Miyake and Friedman, 2012). Therefore, it can be argued that especially for the soccer-specific tasks, there is theoretical support and conceptual overlap. This conceptual overlap indicates a relation between the constructs in accordance with the unity–diversity framework and lends support to the face validity of both soccer-specific tasks assessed in the SoccerBot360.

Given that the general and sport-specific tasks are not highly correlated, the newly developed tasks also seem to differ to some degree from the general cognitive tasks. Thus, it could be discussed to which degree the soccer-specific tasks implemented here, should be considered cognitive tasks and to which degree the motor response might even make it a *motor task*. However, based on the medium correlations between the general and sport-specific tasks, we conclude that the shared variance reflects the cognitive core of the tasks, while the rest of the variance could be attributed to different processes influencing the performance such as perceptual and motor processes relevant for successful performance (i.e., pressing a button vs. passing a ball).

### Limitations

The present project has some methodological limitations. The first is related to the study design of both studies: For each participant, the task in the SoccerBot360 was conducted after the computerized task. This could have potentially resulted in learning effects, which on the one hand were intended to reduce physical exhaustion and prevent injuries in the SoccerBot360 but on the other hand might have biased the results. However, the order of the two computerized and SoccerBot360 tasks were counterbalanced in the main study.

Second, concerning data collection, in the soccer-specific task, the starting point for passing the ball was not always exactly at the same location. However, the SoccerBot360 does not take the time the ball hits the wall as the starting time but rather the time when the ball leaves the foot of the player. Thus, response time measures should not have been affected by slightly differing starting locations of the participants. Third, the response times were inferred from a camera that assesses 120 frames/s. While this way of measuring response times is sufficient for training purposes and for assessing cognitive flexibility as shown in the main study, the diagnostics of inhibition seem to require more precise data. Technically this is feasible and can be implemented in the SoccerBot360 for similar measurements in the future. Importantly, these last two issues did not seem to impact the results considerably when focusing on the variance in response times especially for the soccer-specific flanker task. Fourth, even though both Soccerbot tasks require a soccer-specific motor response (i.e., pass), only the Soccerbot flanker tasks also displays soccer-specific stimuli (i.e., players; see Hadlow et al., 2018). However, including a motor response can be seen as more ecologically valid and presents a first relevant step.

Finally, the follow-up study was conducted with adult soccer players in comparison to the main study which was conducted with younger players. This on the hand increases generalizability of the task but on the other hand it could be argued that those groups are not comparable. Focusing, however, on the process of data analysis, the groups are only analyzed within themselves that thus, a direct comparison is not necessary.

### Future Directions

The results of the present study suggest several future directions for theory and methods in research as well as in the field. In terms of theory, future research should strive to deepen the understanding of which cognitive functions assessed in soccer-specific settings including soccer-specific responses and stimuli are related to soccer expertise. Therefore, future studies should compare different expertise groups and potentially also different age groups (cf. Beavan et al., 2019; Musculus et al., 2019) to infer the relevance of soccer-specific cognitive functions on the route to expertise in soccer. In particular, comparing players of different ages and expertise levels can help clarify which sport-specific cognitive functions are either developing or are indeed expertise related (see e.g., Beavan et al., 2020a; Heilmann et al., 2022b; Turner et al., 2022). By conducting a systematic line of experiments, the predictive validity of the tasks for (future) expertise could also be established further.

Additionally, implementing other methods for validation could be highly interesting from a theoretical perspective. In the flanker task, we found the flanker effect but lower than expected error rates. In order to provide a more sensitive measure, the accuracy of the SoccerBot tasks could be assessed beyond the correct direction (i.e., pass to the left or right) decision. The accuracy of the motor response (i.e., pass) could be better captured by assessing the precision of the pass. In detail, the precision could be captured by determining whether or not the pass hit the goal (i.e., target area). This allows an additional measure about the execution of the motor response which is equally important for the evaluation of inhibition in the soccer context. Further, the goal could even be scaled in size and different target sizes could be presented across trials to obtain a scaled accuracy and precision value for each player. This would be a high added value for the individual player diagnostics and might have direct consequences for training.

Moreover, it is of theoretical and practical relevance to understand how players cognitively adapt to pressure (e.g., Musculus et al., 2018) and the corresponding psychophysiological stress responses (e.g., Lautenbach et al., 2014). When psychophysiological stress is high, for example, at the end of a competitive soccer match, players still have to be able to inhibit and react cognitive flexible. Thus, striving to better understand how psychophysiological stress affects (sport-specific) cognitive functions is warranted (e.g., Lautenbach et al., 2016). This might be studied in the future by manipulating psychophysiological stress, and emotional states, thus targeting “resilient cognition” that is cognitive performance under psychophysiological demands (Walton et al., 2018).

Methodological issues should be discussed in light of practical applicability. To be applicable in an everyday (professional) soccer setting, the soccer-specific cognitive tasks need to be feasible (cf. Beavan et al., 2020b). In particular, a reduction in the number of trials should be aimed at in the future. If the tasks can be reliable with fewer trials this would support the use of soccer-specific cognitive diagnostics in the future. Furthermore, though not every academy would be able (and willing) to invest in technologies such as the Soccerbot360, they could adapt existing diagnostics to their own means (see Murr et al., 2021 for decision-making). Relatedly, soccer clubs, coaches, and sport psychologists working in the field should aim at developing the potential of in-house diagnostics further (Musculus and Lobinger, 2018). In detail, it would make sense to develop shorter parallel versions of the same sport-specific cognitive tasks. Clubs could then establish their own norms and (developmental) benchmarks for sport-specific cognitive functions by enrolling new players in a systematic cognitive diagnostics program and by repeatedly testing the same players, thereby, potentially allowing for talent identification one day. Similarly, a well-established, sport-specific cognitive diagnostics program containing parallel versions could also serve as a tool for quantifying the effects of soccer-specific cognitive training interventions being conducted in the soccer lab or on the field. In this context, longitudinal studies have to be implemented investigating whether superior performance in cognitive paradigms is related to soccer performance on the field and thus can serve as potential predictor for future success (Sakamoto et al., 2018; Van Maarseveen et al., 2018; Beavan et al., 2020b).

Beyond the specific findings of this study, the research field faces the challenge to explain complex sports performance and, potentially, to distinguish the contribution of cognitive and motor processes to performance as well as their interaction with respect to performance. However, cognitive and motor processes in sports actions are so closely intertwined (see e.g., Roca et al., 2013) that one can also ask whether the isolation of both is at all possible—even with specific experimental designs and sensitive dependent variables—and should even be aimed at. Recent developments, especially stemming from the field of embodiment, actually move beyond the separation of cognitive and motor processes and rather focus on the interaction (e.g., Musculus et al., 2021 on embodied planning in climbing; Raab, 2021 on embodied choices).

## Conclusion

In the present project, we aimed to develop soccer-specific inhibition (flanker) and cognitive flexibility (number–letter) tasks and implemented them in the SoccerBot360 and SoccerBot100, thereby, combining the strengths of the expert performance and the cognitive component skills approach. Given the flanker effect and the switch effect found for the soccer-specific tasks, one could argue that the tasks seem to place the same cognitive demands as the general, computerized versions of the tasks. This claim is further supported by the convergent validity demonstrated for cognitive flexibility and, after revision, partially for inhibition. Based on the results from our main study and our follow-up study with the revised flanker task, we would cautiously argue that the first version of the sport-specific flanker task tested in the main study was too easy: The stimuli were players as opposed to arrows, which can be considered highly sport specific and more ecologically valid, but the size of the players was too large. However, the revised sport-specific flanker task can be considered valid for response time parameters, but would need revision to be considered a fully (i.e., also for accuracy) valid measurement tool. The soccer-specific version of the cognitive flexibility task showed convergent validity with the computerized version and can be used for diagnostic purposes. Together, the sport-specific tasks contribute applied and theoretical added value to cognitive diagnostics in soccer which we hope will be fruitful in the future for players, coaches, and researchers alike.

## Data Availability Statement

The raw data supporting the conclusions of this article will be made available by the authors, without undue reservation.

## Ethics Statement

The studies involving human participants were reviewed and approved by the German Sport University Cologne (No: 056/2019). Written informed consent to participate in this study was provided by the participants legal guardian/next of kin. Written informed consent was obtained from the individual(s) for the publication of any potentially identifiable images or data included in this article.

## Author Contributions

LM, MP, and PW: idea. FL and LM: conceptualization, first draft, and supervision. LM, FL, SK, and MR: planning of the study, revision, literature research, and data analysis. NG, AB, and MP: data collection coordination and participant recruitment. SK and MR: data collection. PW, NG, AB, and MP: resources. SK, MR, and PW: data preparation. All authors contributed to the article and approved the submitted version.

## Conflict of Interest

NG and AB are employed by RasenBallsport Leipzig GmbH, MP is employed by FC Bayern München AG and was employed by RasenBallsport Leipzig GmbH, and MR is employed by VfB Stuttgart 1893 AG, however, did not work there while the research was conducted. Finally, PW was employed by Umbrella Software GmbH during the time of conducting the first experiment. The remaining authors declare that the research was conducted in the absence of any commercial or financial relationships that could be construed as a potential conflict of interest.

## Publisher’s Note

All claims expressed in this article are solely those of the authors and do not necessarily represent those of their affiliated organizations, or those of the publisher, the editors and the reviewers. Any product that may be evaluated in this article, or claim that may be made by its manufacturer, is not guaranteed or endorsed by the publisher.
