# Supplementary material for: An Assist for Cognitive Diagnostics in Soccer: Two Valid Tasks Measuring Inhibition and Cognitive Flexibility in a Soccer-Specific Setting With a Soccer-Specific Motor Response
Source: Front Psychol. 2022 Mar 31;13:867849. doi: 10.3389/fpsyg.2022.867849 (PMC9009540; doi:10.3389/fpsyg.2022.867849)
Supplement: Supplementary file 1 [file Table_1.DOCX]

**Supplemental Material**

**A) Comparison of General Computer Tasks and Soccer-Specific Soccerbot Tasks (for both Flanker task and Number-letter task)**

**Table A.1**

*Methodological Description of the Tasks*

| Variable | Flanker task for inhibition | | Number–letter task for cognitive flexibility | |
| --- | --- | --- | --- | --- |
|  | General | Soccer-specific | General | Soccer-specific |
| No. practice trials | 4 (2 congruent, 2 incongruent) | 4 (2 congruent, 2 incongruent) | 16 (number only), 16 (letter only), 16 (number–letter combined) | 4 (number only), 4 (letter only), 16 (number–letter combined) |
| No. test trials | 144 (96 congruent, 48 incongruent) | 108 (72 congruent, 36 incongruent) | 128 (64 switch trials, 64 no-switch trials) | 112 (56 switch trials, 56 no-switch trials) |
| No. test blocks (trials per block) | 2 (72) | 2 (54) | 2 (64) | 2 (56) |
| Break between blocks | 30 s | 30 s | Self-paced | 30 s |
| Response correspondence | Fingertip on keyboard (white button “E” for target arrow pointing to the left; black button “I” for target arrow pointing to the right) | Pass to left goal for target player facing left or pass to right goal for target player facing right | Fingertip on keyboard (white button “E” for consonants and even numbers; black button “I” for vowels and odd numbers) | Pass to left goal for consonants and even numbers or pass to right goal for vowels and odd numbers |
| Stimulus presentation | 5 arrows (middle arrow as target arrow; 2 × 2 flanker arrows) | 5 soccer players (middle player as target player; 2 × 2 flanker players) | Letter–number pair in a 2 × 2 matrix in front of white background | Letter–number-pair in a 2 × 2 matrix in front of green background |
| Randomization of presented stimuli | Yes | No | Yes | No |
| Fixator (fixation duration) | Yellow ★ (1,000 ms) | Black + (1,000 ms) | Yellow quadrant at the beginning of each block (2,000 ms) | Yellow quadrant at the beginning of each block (2,000 ms) |
| Response–stimulus interval | 1,000 ms | 1,000 ms | 150 ms (correct response)  1,500 ms (incorrect response) | 1,000 ms |
| Response time out | 1,750 ms | No | No | No |
| Cronbach’s Alpha  (reaction time) | Congruent: .93  Incongruent: .89 | Congruent: .96  Incongruent: .95 | Switch: .95  No-Switch: .93 | Switch: .94  No-Switch: .95 |
| Split-Half Reliability  (reaction time) | Congruent: .89  Incongruent: .86 | Congruent: .98  Incongruent: .96 | Switch: .82  No-Switch: .82 | Switch: .92  No-Switch: .90 |

**B) Comparisons to Previous Inhibition and Cognitive Flexibility Tasks**

**Table A.2**

*Detailed Comparisons to Previous Cognitive Tasks (i.e., Flanker task, number-letter task)*

| **Author** | **Participants** | **Objectives** | **Flanker Task Design** |
| --- | --- | --- | --- |
| Howard et al. (2014) | 115 students & 120 undergraduates’ | Evaluating Inhibition models for diversity and development of attentional inhibition | Trials: 60 (30 congruent & 30 incongruent randomly selected)  Response time out: 2000ms  Fixation duration: 250ms fixation cross |
| Krenn et al. (2018) | 184 Australian elite athletes  (*M_age_* = 23.2) | examine differences in executive functions depending on sport type | Trials: 108 (72 congruent & 36 incongruent)  Response-stimulus interval: 1000ms  Inter-trial interval: 500, 750 or 1000ms (randomized & counterbalanced) |
| Ridderinkhof & van der Molen (1995) | Males: 5-6 years *(n = 10*), 7-9 years (*n = 18*), 10-12 years (*n = 17*), adults *(n = 17*) | age- related changes in visual selective attention | Trials: 264 (88 congruent, 88 incongruent, 88 neutral)  Inter-trial interval: 2500 and 3500m (varied randomly)  Stimuli presented for 1360ms |
| Stins et al. (2007) | 137 12-year- old children | ability to deal with response conflict & performance variation on response interference tasks by genetic variation | Trials: 80 (40 congruent & 40 incongruent)  Fixation duration: 500ms white fixation cross  Inter-trial interval: 800ms |
| Wu et al. (2011) | 48 preadolescent children  (*M_age_* = 10.1) | Relationship between aerobic fitness and cognitive variability in preadolescent Children | Trials: 200 (100 congruent & 100 incongruent)  Stimuli presented for 200ms  Inter-trial interval: 1700ms |

| **Author** | **Participants** | **Objectives** | **Number-Letter Task Conditions** |
| --- | --- | --- | --- |
| Benedek et al. (2014) | 230 students  (*M_age_* = 23.0) | Common and differential relationship of executive functions to fluid intelligence and creativity. | Letter-Trials: 24  Number-Trials: 24  Combined Trials: 72 (3 blocks)  Response-Stimulus Interval: not reported |
| Gamboz, Borella and Brandimonté (2009) | 40 younger adults (*M_age_* = 29.2)  40 older adults (*M_age_* = 67.8) | Explore how local and global switching, inhibition and working memory, assessed through the Number–Letter, the Stop Signal and the Reading Span tasks, relate to older adults’ performance in the WCST. | Letter Trials: 64  Number-Trials: 64  Combined Trials: 128 (one block)  Inter-Trial-Interval: 200 ms |
| Miyake et al (2000) | 137 undergraduate students  (no mean age reported) | Examine the separability of three often postulated executive functions (Shifting, Updating, Inhibition) and their roles in complex “frontal lobe” or “executive” tasks. | Letter-Trials: 32  Number-Trials: 32  Combined Trials: 128  Response-Stimulus-Interval: 150 ms |
| Parong (2015) | 89 physical active students  (*M_age_* = 21.8) | Effect of Exergames (cognitive engagement and physical activity) on executive functions | Letter Trials: 24  Number-Trials: 24  Trials: 96 trials (one block)  Response-Stimulus-Interval: not reported |
| Purić & Pavlović  (2012) | 62 undergraduate psychology students  (no mean age reported) | Explore the executive function of shifting and its relation to intelligence and personality constructs. | Practice Trials: 32  Trials: 128  Inter-Stimulus-Interval: 150 ms |
| Steinberg & Doppelmayr (2017) | 20 certified recreational divers  (*M_age_* = 30.0) | Comparison of executive functions on land and in water (5-m depth and 20-m depth) | Letter Trials: 16  Number-Trials: 16  Combined Trials: 32 (two blocks)  Response-Stimulus-Interval: 300 – 500ms |

**C) Detailed Description of the Soccer-specific Tasks in the SoccerBot360**

**Figure A.1**

*SoccerBot360*


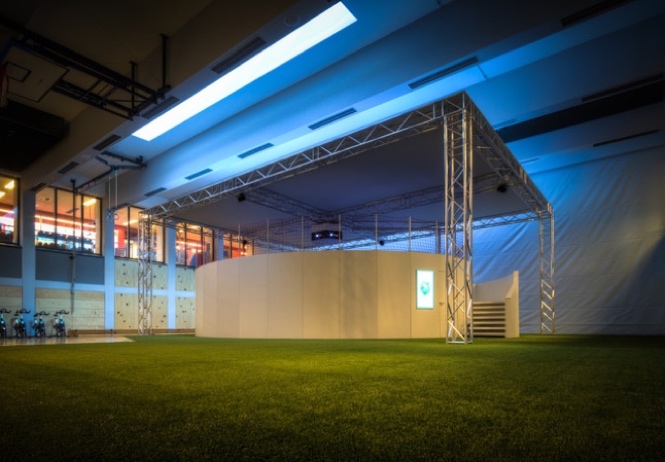

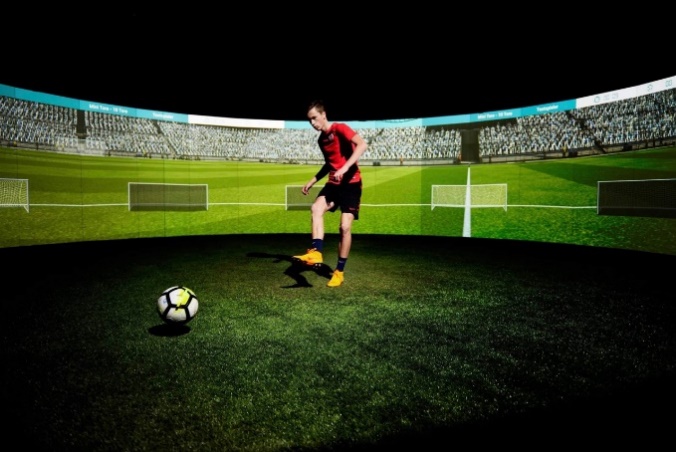


**Soccer-specific tasks in the SoccerBot360**

**Soccer-specific flanker task.** The adapted version of the Flanker task was presented to the participants in the SoccerBot360. Instead of arrows, 5 soccer players were presented from the side. In accordance to the Flanker task, the target player was in the middle surrounded by two players on each side. Each soccer player was wearing the same clothes (i.e., black shirt and short with one red strip) and had a height of 1.80m, the feet were 0.83m wide, arms 0.50m and the heads of the players 0.22m. The distances between the players were 0.79m to the head, 0.50m to the hand and 0.18m to the feet. The distance from the goals to the middle of the target stimuli player was 2.50m.

**Soccer-specific number-letter task.** The adapted version of the number-letter task was presented to the participants in the SoccerBot360. The 2x2 matrix was displayed on two of the wall segments with a size of 2x2 meters. The distance of the two goals (width: 2.20 meters, height: 0.95 meters) in which the participants were asked to pass to the matrix was 0.40 meter to the left and right. Therefore, eight of the 52 plates were being used for this task. The presented stimuli had a height of 0.30 meters and a width of 0.33 meters.

**C Data of Soccer-Specific Flanker Task**

**Figure A.2**

*Scatterplots for General and Sport-Specific Flanker Task for Congruent and Incongruent Trials*


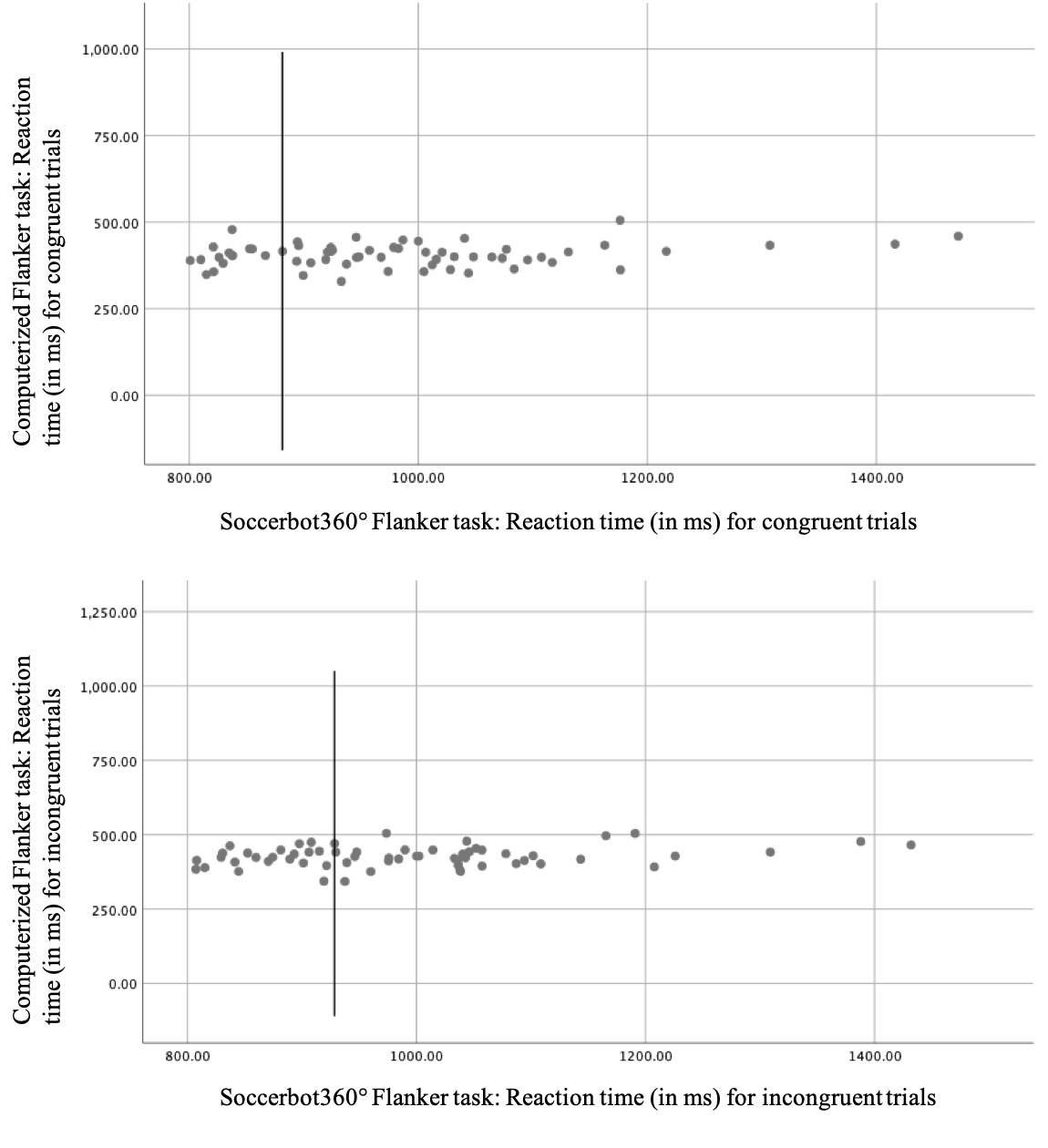


**Table A.3**

Comparison of the applied stimuli dimensions for the soccer-specific flanker tasks

| Stimuli dimensions | SoccerBot360 (Study 1) | SoccerBot100 (Study 2) |
| --- | --- | --- |
| **Height** | 1.80 m | 0.38 m |
| **Width** |  |  |
| Feet | 0.83 m | 0.20 m |
| Arms | 0.50 m | 0.12 m |
| Head | 0.22 m | 0.70 m |
| **Distances** |  |  |
| Head to Head | 0.79 m | 0.14 m |
| Hand to Hand | 0.50 m | 0.10 m |
| Feet to Feet | 0.18 m | 0.50 m |
| Goal to Target Player | 2.50 m | 1.20 m |
